# mSphere of Influence: Decoding Transcriptional Regulatory Networks To Illuminate the Mechanisms of Microbial Pathogenicity

**DOI:** 10.1128/mSphere.00917-19

**Published:** 2020-01-08

**Authors:** Sadri Znaidi

**Affiliations:** a Institut Pasteur de Tunis, University of Tunis El Manar, Laboratoire de Microbiologie Moléculaire, Vaccinologie et Développement Biotechnologique, Tunis, Tunisia; b Institut Pasteur, INRA, Département Mycologie, Unité Biologie et Pathogénicité Fongiques, Paris, France

**Keywords:** *Candida albicans*, genomics, host-pathogen interactions, iron metabolism, transcription factors

## Abstract

Sadri Znaidi works in the field of molecular mycology with a focus on functional genomics in Candida albicans. In this mSphere of Influence article, he reflects on how the paper “An iron homeostasis regulatory circuit with reciprocal roles in Candida albicans commensalism and pathogenesis” by Chen et al. (C. Chen, K. Pande, S. D. French, B. B. Tuch, and S. M. Noble, Cell Host Microbe 10:118–135, 2011, https://doi.org/10.1016/j.chom.2011.07.005) made an impact on his research on how transcriptional regulatory networks function to control C. albicans’ ability to efficiently interact with the host environment.

## Abstract (precis)

Sadri Znaidi works in the field of molecular mycology with a focus on functional genomics in Candida albicans. In this mSphere of Influence article, he reflects on how the paper “An iron homeostasis regulatory circuit with reciprocal roles in Candida albicans commensalism and pathogenesis” by Chen et al. (C. Chen, K. Pande, S. D. French, B. B. Tuch, and S. M.

## COMMENTARY

The advent of functional genomics and systems biology has equipped us with the tools allowing us to decipher the biological circuitries that govern the expression of traits enabling microbial pathogens to efficiently thrive in the host environment. The armamentarium of virulence mechanisms used by pathogens to infect the human body is extensive, ranging from toxin release and tissue invasion (1, 2) to deprivation of host nutrients (3) and hijacking of immune cells (4). The opportunistic yeast Candida albicans adopts two lifestyles, a commensal lifestyle whereby it acts as a bystander, colonizing body organs without causing harmful effects, and a “pathogenic identity” in which it fully expresses virulence traits to cause disease. This duality was elegantly featured in the paper “An iron homeostasis regulatory circuit with reciprocal roles in Candida albicans commensalism and pathogenesis,” by Chen et al. in 2011 (5), where the authors decode the transcriptional network that controls the ability of C. albicans to acquire iron (Sef1 regulon) while thriving as a pathogen in the iron-scarce environment of the bloodstream and map the circuitries that protect C. albicans from toxicity of high iron levels (Sfu1 regulon) encountered in the gastrointestinal (GI) tract, where C. albicans can thrive as a commensal. This work suggested that the C. albicans genome encodes two main genetic switches, i.e., transcription factors (TFs) Sfu1 and Sef1, allowing C. albicans to perfectly adapt to constraints imposed by iron availability in the GI tract and in the bloodstream, respectively. Such a *modus operandi* exemplifies how the evolutionary process remodeled the behavior of microbes that transition from a commensal to a pathogenic state and highlights the impact of decision-making proteins like TFs on their remarkable adaptation to disparate host niches. This study by the Suzanne Noble group was a source of inspiration to my research, prompting me to further investigate the consequences of genetically perturbing TFs on C. albicans’ phenotypic traits pertaining to pathogenicity and commensalism and comprehensively map the associated transcriptional circuitries using genome-wide expression and location technologies. Future perspectives in this particular field, and in microbial systems biology in general, will certainly focus on generating a global landscape of TF networks that impact on key fitness attributes underlying the pathogenic and commensal facets of opportunistic microbes.

The Chen et al. paper (5) built on previous findings where *SEF1* showed up among a subset of genes whose deletion altered C. albicans infectivity in a murine model of systemic infection (6) and its ability to grow on iron-depleted medium (7). The fact that *SEF1* encodes a TF suggested that it may act as a transcriptional regulator of iron uptake; however, the prevailing model based on the rich literature from Saccharomyces cerevisiae and Schizosaccharomyces pombe was in favor of transcriptional control through positive (S. cerevisiae AFT-type regulators Aft1/Aft2) or negative (S. pombe GATA-type regulator Fep1) regulation (8, 9). The C. albicans genome encodes one homolog of each TF, respectively, named Aft2 and Sfu1 (10, 11). However, while Sfu1 is clearly involved in transcriptional repression of iron uptake genes (10), the role of Aft2 appears to be somewhat pleiotropic (12), and its function in C. albicans could have been modified. The authors combined chromatin immunoprecipitation (ChIP) and transcript profiling to decipher the regulatory network of Sef1 and Sfu1, together with Hap43, a conserved regulator of iron utilization genes (13). They found that the three TFs form a tightly knit circuitry, in which Sef1 clearly acts as an activator of iron uptake genes, whereas Sfu1 and Hap43 act mainly as negative regulators of iron uptake and iron utilization genes, respectively (5). These findings are striking, as they point to the occurrence of evolutionary remodeling of the circuitry controlling iron homeostasis in C. albicans, a process also termed “transcriptional rewiring” (14). The authors further emphasized the functional importance of the rewired circuitry. They showed that the *sef1^−/−^* and *sfu1^−/−^* mutants failed to transcriptionally induce and repress iron uptake genes in human plasma and in the GI tract and were inefficient at causing disease in a systemic infection model and at colonizing the murine gut, respectively, pointing to functional specificities associated with the two host niches. The results from animal experiments are fascinating, clearly demonstrating the specialization of the two genetic switches in the expression of pathogenic (iron acquisition from the host) and commensal (protection from iron toxicity) traits of C. albicans when it thrives in the bloodstream and in the gut.

The Chen et al. paper (5) brought me straight back to my M.Sc. and Ph.D. years. In 2004, while preparing to move to Montreal, Canada, to start working with Martine Raymond on transcriptional regulation in C. albicans, I deposited the final version of my Master’s thesis on the characterization of the iron regulator Fep1 (15); the last two paragraphs of my thesis commented on the following question: how close is transcriptional regulation of iron uptake in fission yeast to that in C. albicans? This question was based on the relatively high level of homology that we observed between the deduced amino acid sequences of *fep1*^+^ and *SFU1*. Later on, the Labbé lab found that heterologous expression of C. albicans Sfu1 in S. pombe produced a functional protein that was able to turn off iron uptake genes in fission yeast (16). This study could have been a great starting point for comparative analyses of the transcriptional circuits that control iron homeostasis in the two yeast species, but the technologies allowing us to perform them were not routinely implemented yet. At that time, the C. albicans genome sequence had just been published (17), and many members of the *Candida* community started contributing to its annotation (18, 19). The excitement for doing functional genomics in C. albicans was mind-blowing at that time, and many labs—including my Ph.D. lab—started using and combining the ChIP-chip (ChIP with microarray technology) and transcriptomics technologies to map transcriptional regulatory networks (20–22). The Chen et al. paper naturally influenced my thinking for the following reasons. (i) It elegantly addressed one of the questions I was eager to answer during my early years in research. (ii) It exemplified the powerful use of genome-wide expression and location technologies to explain how transcriptional circuits are built, connected, and remodeled in nonpathogenic versus pathogenic yeast species. (iii) It fostered looking deeper into conditions that mimic the host niches in which C. albicans thrives. (iv) It used relevant animal models to learn more about the fascinating behavior of this opportunistic yeast. This paper definitely laid the groundwork for implementing future large-scale approaches, such as systematic genetic perturbation of TFs combined with high-throughput phenotyping and ChIP-sequencing (ChIP-Seq) or transcriptome sequencing (RNA-Seq), providing a bird’s eye view of the transcriptional circuitries operating in microbial species that alternate between commensal and pathogenic lifestyles.

The literature has been quite rich in discoveries since publication of the Chen et al. study (5). We currently know that Sef1 exerts its function through iron-dependent nuclear localization, a process orchestrated by its physical association with Sfu1 in the cytoplasm, when iron is abundant, and phosphorylation by Ssn3 cyclin-dependent kinase and nuclear localization, when iron is scarce (23). More recently, the Aberdeen Fungal Group showed that *SEF1* acts as a determinant of C. albicans’ immune evasion capacity by strongly masking β-glucan (24), consistent with its role in C. albicans’ virulence. Now, what happens in other *Candida* species? Work by the Hube lab provided further evidence that the evolution of iron sensing/uptake regulatory networks is quite flexible from one species to another (25). While C. albicans intercalated Sef1 into an ancient circuit (i.e., employed by S. pombe), C. glabrata apparently combined the systems used in C. albicans (Sef1) and S. cerevisiae (Aft1) to control iron homeostasis (25). Still, like in S. cerevisiae, Aft1 acts as the major regulator of iron uptake in C. glabrata, whereas Sef1 appears to control iron consumption processes (25). Clearly, future work will reveal quite surprising discoveries in the field, further illuminating the fascinating strategies used by microbes in the arms race for nutrient iron during host-pathogen interactions.
